# Safety and Efficacy of Hizentra® Following Pediatric Hematopoietic Cell Transplant for Treatment of Primary Immunodeficiencies

**DOI:** 10.1007/s10875-023-01482-y

**Published:** 2023-06-02

**Authors:** Niraj C. Patel, Troy Torgerson, Monica S. Thakar, M. Elizabeth M. Younger, Panida Sriaroon, Tamara C. Pozos, Rebecca H. Buckley, David Morris, Diana Vilkama, Jennifer Heimall

**Affiliations:** 1 Department of Pediatrics, Division of Allergy and Immunology, Duke University, Durham, NC USA; 2 Atrium Health, Charlotte, NC USA; 3 Allen Institute for Immunology, Seattle, WA USA; 4 Clinical Research Division, Fred Hutchinson Cancer Center, Seattle, WA USA; 5 Department of Pediatrics, University of Washington, Seattle, WA USA; 6 Division of Allergy, Immunology and Rheumatology, Johns Hopkins University School of Medicine, Baltimore, MD USA; 7 Division of Allergy and Immunology, University of South Florida, Tampa, FL USA; 8 Department of Clinical Immunology, Children’s Minnesota, Minneapolis, MN USA; 9 Webbwrites, Durham, USA; 10 Division of Allergy and Immunology, Children’s Hospital of Philadelphia, Philadelphia, PA USA

**Keywords:** Subcutaneous immunoglobulin, Hematopoietic cell transplant, Inborn errors of immunity, Primary immunodeficiency diseases

## Abstract

Primary immunodeficiency disease (PIDD) comprises a group of disorders of immune function. Some of the most severe PIDD can be treated with hematopoietic cell transplant (HCT). Hizentra® is a 20% liquid IgG product approved for subcutaneous administration in adults and children greater than 2 years of age with PIDD-associated antibody deficiency. Limited information is available on the use of Hizentra® in children following HCT for PIDD. A multicenter retrospective chart review demonstrated 37 infants and children (median age 70.1 [range 12.0 to 176.4] months) with PIDD treated by HCT who received Hizentra® infusions over a median duration of 31 (range 4–96) months post-transplant. The most common indication for HCT was IL2RG SCID (*n* = 16). Thirty-two patients switched from IVIG to SCIG administration, due to one or more of the following reasons: patient/caregiver (*n* = 17) or physician (*n* = 12) preference, discontinuation of central venous catheter (*n* = 16), desire for home infusion (*n* = 12), improved IgG serum levels following lower levels on IVIG (*n* = 10), and loss of venous access (*n* = 8). Serious bacterial infections occurred at a rate of 0.041 per patient-year while on therapy. Weight percentile increased by a mean of 16% during the observation period, with females demonstrating the largest gains. Mild local reactions were observed in 24%; 76% had no local reactions. One serious adverse event (death from sepsis) was reported. Hizentra® was discontinued in 15 (41%) patients, most commonly due to recovery of B cell function (*n* = 11). These data demonstrate that Hizentra® is a safe and effective option in children who have received HCT for PIDD.

## Introduction

Primary immunodeficiency diseases (PIDD), also known as inborn errors of immunity, are a group of disorders that result in an increased susceptibility to infections, autoinflammatory disease, and cancer [1–5]. Immunoglobulin G (IgG) replacement therapy is indicated for the treatment of patients with PIDD associated with defects in the ability to produce normal quantity and quality of endogenous immunoglobulin. Intravenously-administered IgG (IVIG) is commonly administered every 3–4 weeks but is associated with systemic side effects in up to 15% of infusions, relies on venous access, and must be administered by a healthcare provider [6–11]. Subcutaneously-administered IgG (SCIG) formulations are similarly efficacious to IVIG in prevention of infection, but result in fewer systemic side effects (< 1% of infusions), and can be infused at home [12–18].

Hematopoietic cell transplant (HCT) remains the treatment of choice for many PIDDs, such as severe combined immunodeficiency (SCID) and hemophagocytic lymphohistiocytosis (HLH). Recommended use of IgG pre and post-HCT has been described [19, 20] and included poor or incomplete immunity due to pre-HCT status or mixed T and/or B cell chimerism [21] and prevention and treatment of infections [22].

Reasons for requiring IVIG in the immediate peri-transplant setting may include the presence of a central venous catheter, frequent clinic follow-up visits necessitating blood draws, and frequent infusions of IV medications and blood products. The role of SCIG in the post-transplant setting is less clear. Limited information on the use of SCIG is available for patients who have received HCT for malignancy [23–26], or very young pediatric patients with PIDD [27]. The nationwide implementation of newborn screening for SCID, a PIDD treated with HCT, has led to increased identification of newborns with SCID [28]. In these infants, early HCT before 3.5 months of age and prior to development of infection is associated with the best survival. These newborns may benefit from supportive care with SCIG before and also after transplant while awaiting B cell function to recover [29, 30]. Unfortunately, some patients with PIDD do not recover B cell function following HCT, and these patients may also be candidates for longer term post-transplant SCIG [31].

Hizentra® is a 20% liquid SCIG formulation approved for use by adults and children greater than 2 years of age with PIDD [32–34] and has also been reported to be safe and effective in children less than 2 years of age [27, 35]. We assessed the safety and efficacy of Hizentra® in pediatric subjects pre- and post-HCT. Because HCT is frequently associated with central venous catheter use, we assessed the reasons for switch from IVIG to SCIG. Neutropenia during the HCT period is a major risk factor for infection including cellulitis [36]. Because subcutaneous administration of immunoglobulin could increase risk of infection due to local trauma, neutrophil counts were evaluated during the HCT period.

## Methods

A multicenter retrospective chart review was performed to identify children who received HCT for an underlying PIDD diagnosis and were treated with Hizentra® replacement therapy for supportive care post-transplant. The study was approved by the Institutional Review Board at all participating institutions. Patients were included if they received more than one dose of Hizentra® after HCT (and prior to HCT in some patients), including those on other Ig replacement (IVIG or SCIG) for their initial therapy. A 1:1 conversion factor was used when converting patients receiving IVIG to SCIG. Infusions in patients older than 18 years of age were not examined. Exclusion criteria included patients with protein losing conditions such as lymphangiectasias, nephrosis, or protein losing enteropathies. Study data were collected and managed using Research Electronic Data Capture (REDCap) electronic data capture tools hosted at Carolinas Medical Center [37]. REDCap is a secure, web-based application designed to support data acquisition for research studies. Descriptive statistics only are reported. Primary outcomes including efficacy and safety of treatment were based on rates of serious bacterial infections (SBIs), non-SBIs, and adverse events (AEs). Rates of SBIs and non-SBIs were reported for study subjects prior to receiving Hizentra®. Reported SBIs included bacteremia/sepsis, bacterial meningitis, bacterial pneumonia, osteomyelitis/septic arthritis, and visceral abscesses (liver, lung, and brain) based on pre-defined criteria adjusted to pediatric patients (FDA Guidance for Industry 2008). Information about AEs was collected, including local reactions at the infusion site. Other study assessments included serum IgG levels, volume of infusion, number of infusion sites, duration of infusions, neutrophil counts, and height and weight measurements during treatment period.

Rate of infusions was assessed among patients receiving Hizentra® by pump or manual delivery, and a cut-off value of > 20 mL/h was used for rapid delivery based on previously published studies [16, 38, 39]. Characteristics around HCT, central venous catheter history, and complications post-HCT were collected. Reasons for switch from IVIG to SCIG were determined. Weight and body length, previously shown to be associated with improvement in growth percentiles during SCIG treatment [25], were recorded at the start and end of treatment. Only patients with values at both time points were included. Results were provided for all patients and difference in mean weight, and length percentile was calculated using a *t*-test.

## Results

### Study Population

Thirty-seven children with PIDD from 8 centers were available for review. Patient demographics and underlying PIDD diagnoses are shown in Table 1. Thirty-two (86%) were males, and the most common race was white (*n* = 24 patients, 65%). The most common indications for HCT included SCID (*n* = 28) in 76% of children, while the remainder (*n* = 9, 14%) received HCT for various indications including hemophagocytic lymphohistiocytosis, X-linked chronic granulomatous disease, cytotoxic T-lymphocyte-associated protein-4 (CTLA-4) haploinsufficiency, hyperimmunoglobulin M syndrome, signal transducer and activator of transcription 1 (STAT1) gain-of-function (GOF), and 22q11 deletion syndrome.

**Table 1:** Demographics, characteristics, and diagnosis for 37 transplanted PIDD children at the start of Hizentra®

Patient characteristic	Patients total, No. (%)
Gender, *n* (%)	
Male	32 (86)
Female	5 (14)
Race, *n* (%)	
White	24 (65)
Asian	5 (14)
Hispanic	2 (5)
Black or African American	2 (5)
Other	4 (11)
Age and growth parameters	
Age, months, mean (range)	70.1 (12.0–176.4)
Height, cm, mean (range)	78.2 (49.0–124.0)
Weight, kg, mean (range)	11.2 (3.2–38.0)
Diagnosis, *n* (%)	
SCID	28 (75.7)
X-linked	16 (43.2)
ADA	3 (8.1)
RAG2	2 (5.4)
IL7R	2 (5.4)
JAK3	2 (5.4)
Artemis	1 (2.7)
Other NK^+^SCID	2 (5.4)
Other	9 (24.3)
Hemophagocytic lymphohistiocytosis	3 (8.1)
X-linked CGD	2 (5.4)
CTLA-4 haploinsufficiency	1 (2.7)
Hyperimmunoglobulin M syndrome	1 (2.7)
STAT1 GOF	1 (2.7)
22q11 deletion syndrome	1 (2.7)
HCT conditioning	
None	18(49)
Myeloablative	4 (11)
Reduced intensity	12 (32)
Serotherapy only	3 (8)
Donor	
Bone marrow	23 (62)
PBSC	8 (22)
Cord	6 (16)
Acute GVHD	
Grades 1–2	11 (30)
Grade 3–4	7 (19)
Chronic GVHD	
Mild	6 (16)
Moderate	2 (5)
Severe	1 (2.5)

*ADA*, adenosine deaminase deficiency; *CGD*, chronic granulomatous disease; *CTLA-4*, cytotoxic T-lymphocyte-associated protein-4; *GVHD*, graft-versus-host disease; *HCT*, hematopoietic cell transplant; *IL7R*, interleukin 7 receptor; *JAK3*, Janus kinase 3; *NK*, natural killer; *PBSC*, peripheral blood stem cells; *PIDD*, primary immunodeficiency diseases; *RAG*, recombination activating; *SCID,* severe combined immunodeficiency; *STAT1 GOF*, signal transducer and activator of transcription 1 gain-of-function

The median age at transplant was 4 (range 0.5–111) months. The majority of the cohort received either no conditioning (49%) or serotherapy only (8%) prior to HCT. Myeloablative conditioning was used in 11%, while reduced intensity conditioning was administered to 32%. With regard to stem cell source, 62% received bone marrow, 22% received peripheral blood stem cells (PBSC), and 16% children received cord blood (Table 1). Graft versus host disease (GVHD) prophylaxis was used in 22 patients, and pharmacologic immunosuppression included one or more of the following: cyclosporine (*n* = 13), mycophenolate mofetil (*n* = 11), tacrolimus (*n* = 8), cyclophosphamide (*n* = 6), methotrexate (*n* = 5), corticosteroid (*n* = 2), and sirolimus (*n* = 1). Ten patients received no GVHD prophylaxis, and 5 patients received T cell-depleted donor bone marrow only. Acute GVHD post-HCT included grades 1–2 in 11 (30%) children and grades 3–4 in 7 (19%) children. Chronic GVHD was mild, moderate, or severe in 6, 2, and 1 child(ren), respectively. The most common site was skin in both acute GVHD (grades 1–2, *n* = 7, grades 3–4, *n* = 7) and chronic skin GVHD (*n* = 8) (Table 1**)**. One patient died at 16 months post-transplant from bacterial sepsis.

### Infusion Parameters

The median age at the start of Hizentra® was 15 months (range 0.5–114), with median start date post-transplant 11 months (range 0.5–87). Patients were observed from 4 to 96 months while receiving infusions every 7–14 days. Three patients started therapy prior to transplant, and an additional six patients started at ≤ 6 months post-HCT. Five patients naïve to immunoglobulin therapy were started on Hizentra® directly, without prior administration of other immunoglobulin products, including IVIG.

Median cumulative monthly dosage was lower for patients receiving SCIG than IVIG. The median SCIG dose was 776 mg/kg/4 weeks, and the median IgG level available in 34 patients was 871 (range 404–1780) mg/dL). Thirty-two patients received IVIG therapy prior to Hizentra® including a median dose of 807 (mg/kg/4 weeks and median trough IgG level of 696 (range 316–1170) mg/dL. Two patients received SCIG (Vivaglobin®, 16% liquid formulation, CSL Behring) prior to Hizentra®.

Hizentra® was administered subcutaneously using infusion pumps (*n* = 31) or manual delivery (*n* = 6). A total of 4107 infusions were administered. The median number of infusions per patient was 111 (range 8–316). Premedication prior to SCIG infusion was utilized in only 1 patient (lidocaine cream). Sites of administration included thigh (62%), thigh and abdomen (14%), buttocks (14%), abdomen (8%), and thigh and arm (2%). The median total treatment volume per patient was 10.4 mL (range 5–45), while median volume per site for all patients was 6.1 mL (range 2.5–22.5 mL). Manual delivery was most often accomplished using a 23-, 25-, or 27-gauge butterfly needle set, and the media volume per site was 3.1 mL (range 1.5–5 mL). Needle length was reported in 18 patients, and the most frequently used size was 6 mm (72%), followed by 4 mm (17%) and 9 mm (11%). In all patients, therapy was administered at home after instruction was completed. A parent or caregiver administered the infusions in 78% patients. The remaining 22% patients received administration by a home care or clinic infusion nurse. Median SCIG infusion time was 67.1 min (range 10–240) for pump entry and 5.2 min (range 3–10) for manual entry, and the average number of infusion sites was 1.7 (range 1 to 2). The median rate (mL/h) was 20.0 (range 5–135) for pump entry and 120 (range 100–200) for manual entry. Rapid infusion (> 20 mL/h) was achieved by 100% of patients in the manual entry group and in 6% of patients in the pump-administered group, with similar infusion volumes in both groups. Calculated infusion rates were 11.3 mL/h/site (range 0.63–67.5) for pump entry and 90 mL/h/site (range 50–200) for manual entry. Serum IgG levels were higher for patients receiving Hizentra® (871 [range 404–1780] mg/dL among 34 patients) compared to those who received IVIG or other SCIG prior to Hizentra® (696 [316–1170] mg/dL in 36 patients), likely related to higher steady-state level related to increased frequency of SCIG compared to IVIG dosing.

### Efficacy and Tolerability

Infections including SBIs and non-SBIs during IVIG or Hizentra® are shown in Table 2. On therapy, SBIs included sepsis/bacteremia (*n* = 2) and pneumonia (*n* = 3); the incidence of SBIs was 0.041 infections per patient-year. Non-SBIs during the same time period included cellulitis in three patients (central venous catheter site [*n* = 2], gastrostomy tube site [*n* = 1]), central line-associated blood stream infection in two patients, parainfluenza bronchiolitis in two patients, and one patient each with cytomegalovirus viremia, Enterobacter gastroenteritis, *Clostridium difficile* gastroenteritis, or fever without a source. The incidence of non-SBIs was 0.082 infections per patient-year. In 32 patients who received IVIG prior to starting Hizentra®, the incidence of SBIs and non-SBIs were 0.247 and 0.71 infections per patient-year, respectively.

**Table 2:** Comparison of number and rate of infections per patient-year occurring during treatment prior to and during Hizentra®

	Prior to Hizentra®*		Hizentra®	
Infection type	Total number of infections	Rate of infections per patient-year	Total number of infections	Rate of infections per patient-year
Serious bacterial infections				
Bacteremia/sepsis	8	0.220	2	0.16
Bacterial meningitis	0	0	0	0
Pneumonia, bacterial	1	0.027	3	0.025
Osteomyelitis/septic arthritis	0	0	0	0
Visceral abscess (liver, lung, brain)	0	0	0	0
Total	10	0.247	5	0.041
Non-serious infections				
Urinary tract infection	1	0.027	0	0
Pneumonia, viral	1	0.027	0	0.03
Ear infection	1	0.027	0	0.03
Gastroenteritis	1	.027	2	0.06
Abscess/cellulitis	1	.027	2	0.04
Bronchiolitis/tracheitis	4	.11	2	0.10
Viremia (CMV, EBV)	5	0.14	1	0
Lymphadenitis	1	0.027	0	0
Fever	0	0	1	0
Other^#^	11	0.30	2	0
Total	26	0.71	10	0.082

*Includes 37 patients who received IVIG (*n* = 32) or SCIG other than Hizentra® (*n* = 2)

^#^Other infections included central line-associated blood stream infection (*n* = 12: 10 received IVIG, 2 received Hizentra®) and pneumotosis (*n* = 1, IVIG)

The median absolute neutrophil count (ANC) prior to transplant in 35 patients was 4214 (range 0–12,410) cells/µL. The median duration of neutropenia, defined as ANC < 1.5 × 10^3^ cells/µL after HCT, was 7.25 months (range 0.25–36) with neutropenia lasting < 6 months (*n* = 16), ≥ 6–12 months (*n* = 9), and ≥ 12 months (*n* = 10). Two patients did not experience any neutropenia after HCT. Nine patients were neutropenic over the course of administration of Hizentra®, having a median ANC 918 (range 71–1,480) cells/µL, lasting up to 37 months after initiation. There were no episodes of cellulitis associated with infusion sites during this period, although concurrent administration of antibiotic prophylaxis was not examined. While platelet counts were not assessed, bleeding was not reported as a complication of SCIG therapy.

Most (76%) patients experienced no local AEs (Table 3). Local reactions were mostly mild and observed in 9 (24%) children. Excluding local reactions, other AEs included one patient each with pyrexia, headache, rash, or diarrhea. In patients with acute GVHD of the skin (grades 1–2, *n* = 7; grades 3–4, *n* = 7) and chronic skin GVHD (*n* = 8), no adverse events were associated with SCIG treatment. One SAE (death) occurred in a patient with sepsis who at the time of death had no indwelling catheter, was not neutropenic, normal mitogen studies, and had the following donor chimerism: T cells (99%), B cells (2%), and myeloid (2%).

**Table 3:** Summary of adverse events of 37 HSCT children

Adverse event	IVIG^a^, No. (%)	Hizentra®, No. (%)
None	20 (59)	28 (76)
Any adverse event^b^	14 (41)	9 (24)
Local reaction^c^	0 (0)	9 (24)
Pyrexia	3 (09	1 (3)
Hypotension	2 (6)	0(0)
Hives	1(3)	0(0)
Headache	1 (3)	1 (3)
Rash	(0)	1 (3)
Diarrhea	0 (0)	1 (3)
Abdominal pain	1 (3)	0 (0)
Upper respiratory tract infection	1 (3)	0 (0)
Flushing	1 (3)	0 (0)

^a^IVIG adverse events in 32 patients (5 IgG naïve patients were started on Hizentra®)

^b^Multiple adverse events occurred in some patients

^c^Based on 16 MedDRA preferred terms

### Growth Parameters

Weight and length percentiles were assessed at the beginning and end of the treatment observation period. There was an increase in overall weight percentile with a mean increase of 16% [range 1–99%] from 36 to 52% over the observation period (mean 31 months). Females (*n* = 5) showed a trend towards higher increase in weight percentile with a mean of 35% [range − 41–89%] from 52% at start to 87% at end compared to males (*n* = 32) with a mean of 13% [range − 49–97%] from 33% at start to 47% at end (*p* = 0.18). Length parameters for 37 patients were unchanged for both males and females, with a median of 26% [range 1–96%], both at the start and end of the observation period.

### Switch from IVIG to SCIG

For 32 patients who were on other Ig therapy initially, the indications for switching to Hizentra® are described in Table 4; multiple indications were reported for some patients. These include patient/caregiver (*n* = 17) or physician (*n* = 12) preference, discontinuation of central venous catheter (*n* = 16), desire for home infusion (*n* = 12), improved IgG serum levels following IVIG (*n* = 10), and loss of venous access (*n* = 8), and less common reasons included desire for caregiver administration (*n* = 3), adverse events from IVIG (*n* = 2), repeated central line infection (*n* = 1), and missed days of school (*n* = 1). Hizentra® was discontinued in 15 (41%) patients due to recovery of B cell function (*n* = 11). The average duration of therapy in these patients was 14.4 months [range 4–37]. Other reasons for discontinuation included switch to IVIG related to replacement (following a prior discontinuation) of central venous catheter (*n* = 2), behavioral complaints during SCIG (*n* = 1), and death related to sepsis (*n* = 1). Twenty-two (59%) children remained on Hizentra® due to continued need for replacement therapy related to ongoing B cell dysfunction.

**Table 4:** Reasons for switch from IVIG to Hizentra® for 32 HSCT children*^

Clinical	
Removal of central line	16
Improved Ig serum levels and steady state	10
Difficult intravenous access	8
IVIG adverse events	2
Repeated central line infection	1
Non-clinical	
Patient or caregiver preference for SCIG	17
Home site of care preference	12
Physician preference for SCIG	12
Desire for self-administration and increased independence	3
Financial (insurance)	0
**Other	1

*IVIG*, intravenous immunoglobulin; *SCIG*, subcutaneous immunoglobulin

*Multiple fields can be selected by single subject

^ 5 subjects Ig naïve, not included

**Frequent missed school days

## Discussion

This report is the largest to evaluate the safety and efficacy of SCIG (Hizentra®) in 37 children with PIDD who underwent HCT. On therapy, the study population had an annualized rate of SBIs of 0.041 per patient-year compared to 0.247 per patient-year in 32 patients receiving IVIG prior to the observation period. This rate is similar to those observed in older children and adults on SCIG for PIDD [32–34, 40–42]. Infections occurring shortly after the initiation of IVIG therapy prior to obtaining steady-state IgG levels may have contributed to a higher rate of SBIs during this period, with sepsis/bacteremia responsible for the majority of infections. While the median age at transplant was 4 months, the median start date of Hizentra® was not until 15 months, and patients may have benefitted from being more clinically stable at this stage post-HCT, accounting for differences in SBI rates between IVIG and SCIG. Other factors that may have contributed to differences in SBI rates outside of immunoglobulin administration include differences in immunosuppressive medications, engraftment of T cells, and presence of indwelling catheters associated with risk of central line-associated blood stream infections. Local reactions occurred in 9/37 (24%) children and were mild and transient. This is similar to previously published studies on SCIG management and PIDD which report an overall incidence of adverse events of 7–96% [17, 32, 34, 42–44].

Neutropenia is a major risk factor for infection, including cellulitis, during the pre-engraftment period immediately following HCT [36]. Cellulitis is also a well-known complication from central venous catheter placement, particularly in the same pre-engraftment period [45–47]. Subcutaneous administration of medications during neutropenic periods could increase the risk for cellulitis related to local skin trauma following needle administration [48–50]. In this cohort, there were no episodes of cellulitis, in particular none during periods of neutropenia. Severe GVHD may hamper use of SCIG, as infusions in stiff, noncompliant tissues may not be possible or painful [50, 51]. In our cohort, no adverse events were associated with SCIG treatment in patients with acute GVHD of the skin. This suggests that in patients with mild forms of either cutaneous acute or chronic skin GVHD, SCIG may be well tolerated.

The median duration of IVIG prior to switch to Hizentra® was 10 (range 1–86) months. Among these 32 patients, 9 patients were on IVIG for 3 months or fewer prior to switch. Among the reasons for switch from IVIG to Hizentra®, preference for switch by the patient/caregiver and transition of infusions to a home setting were the most common reasons given. Indeed, it has been reported that families of children who underwent HCT favored infusion at home due to reduced need for clinic visits and increased participation in their child’s treatment [24]. In one study, patient satisfaction scores were higher with SCIG compared to IVIG in adults who underwent HCT [25], similar to published reports on SCIG and patient satisfaction/quality of life in PIDD children and adults [52–55]. In our cohort, there were no treatment discontinuations among SCIG patients, possibly related to low rate of adverse events and higher overall satisfaction with SCIG. Twenty-two (59%) children remained on long-term Hizentra® immunoglobulin replacement post-HCT due to ongoing B cell dysfunction, including 13 (59%) patients who received no conditioning, 8 (36%) patients who received reduced intensity conditioning, and 1 (5%) patient who received myeloablative conditioning.

Weight percentile increased by a mean of 16% during the observation period (average 31 months), with females demonstrating the largest gains (mean 35%). Similar observations have been reported in young children during catch-up growth after a period of high infectious disease burden or inadequate dietary intake [56–60], as well as a smaller cohort of HCT children published by our group [27]. There was no difference in length percentile in either male or female patients. Previous observations suggest stunted growth in children requires recovery in weight before resuming linear growth [56, 57, 60]. Perhaps a longer observation period in the current study would have seen differences in gains in length percentiles. Conversely, complications related to pre- and post-transplant infections and post-transplant complications such as GVHD may have adversely affected growth velocity [61], although observed differences may have been limited due to small sample size. Data from this study support the observation that poor growth seen among HCT patients, most likely related to prior frequent and severe infections and transplant complications, can possibly be reversed with optimal immunoglobulin replacement although other factors including genetics may play a role.

This study was limited by the number of study participants, duration of follow-up, and the retrospective nature of the study. The dosages used for both IVIG and SCIG were higher than normal in some patients, which was left up to the discretion of each investigator but could have been related to the clinical condition of the patient, initial loading dose, low IgG trough levels, or other factors. Conclusions were unable to be drawn regarding outcomes related to specific types of PIDD, conditioning regimens, cell sources, and GVHD prophylaxis due to the small study group. Growth changes observed in this study was limited by lack of a comparison cohort. Findings including low rate of local adverse reactions and improved growth parameter need to be confirmed with prospective studies including larger sample sizes and longer observation periods.

Hizentra® appears effective in preventing infections in children receiving HCT and was well tolerated for periods up to 96 months. These data support use of this therapy in pediatric patients with inborn errors of immunity even in the post-transplant period.

## Data Availability

All data supporting the findings of this study are available within the paper.
